# KINSHIP STRUCTURES FOR LEFT BEHIND OLDER ADULTS IN HIGH OUTMIGRATION CONTEXTS

**DOI:** 10.1093/geroni/igae098.1766

**Published:** 2024-12-31

**Authors:** Amilcar Matos-Moreno, Diego Alburez-Gutierrez, Ivan Williams, Ashton Verdery, Mariana Fernandez, Alexis Santos-Lozada

**Affiliations:** The Pennsylvania State University, San Juan, Puerto Rico; Max Planck Institute for Demographic Research, Germany; Universidad de Buenos Aires, Buenos Aires, Argentina; The Pennsylvania State University, University Park, Pennsylvania, United States; Universidad de La República, Montevideo, Uruguay; The Pennsylvania State University, University Park, Pennsylvania, United States

## Abstract

Migration accelerates population aging in high-outmigration contexts; however, the familial structure of older adults left behind has yet to be studied. Evidence suggests that left behind older adults are at higher risk of reduced support networks and increased caregiving burden. This study aims to estimate the kinship structures of older adults, kinlessness, and the presence of migrant kin in Puerto Rico. Data come from the United Nations World Population Prospects from 1950 to 2021. We created a two-sex time-variant model to estimate the kinship structure of older adults in Puerto Rico for 2021. Further, we created a multistate model to estimate the prevalence of transnational family members and their relationship with older adults. Our models suggest that a person born in 1950 will have, in 2021, on average, 5.22 close family members: 1.3 daughters, 1.2 sons, 1.8 sisters, and 1.5 brothers. As they continue to age, the prevalence of female kin supersedes the male presence in the family. Migration is predominant in descendent kins (daughters and nieces). Adults between 20 to 40 are expected to have 0.30 migrant daughters. Older adults between the ages of 60 and 65 have lost 44% of their daughters to migration a lower estimate when compared to adults of the age of 50 or younger (55%). Models suggest that future generations of Puerto Rican older adults will have an increased presence of transnational family members. Thus, public health strategies must adapt to address the need for “new families” in upcoming generations of older adults.

